# How did the COVID-19 pandemic affect inpatient care for children in Germany? An exploratory analysis based on national hospital discharge data

**DOI:** 10.1186/s12913-023-09929-z

**Published:** 2023-08-31

**Authors:** Dimitra Panteli, Nicole Mauer, Florian Tille, Ulrike Nimptsch

**Affiliations:** 1 Department of Health Care Management, Technische Universität Berlin, Strasse des 17. Juni 135, 10623 Berlin, Germany; 2 European Observatory on Health Systems and Policies, Place Victor Horta 40/30, Brussels, 1060 Belgium; 3 European Observatory on Health Systems and Policies, London School of Economics and Political Science, Cowdray House, London, WC2A 2AE UK

**Keywords:** COVID-19, Coronavirus, Paediatric, Child hospitalisations, Hospital admissions, Discharge data, Germany, Appendicitis, Acute lymphoblastic leukaemia, Tonsillectomy, Adenoidectomy

## Abstract

**Background:**

The delivery of health services around the world faced considerable disruptions during the COVID-19 pandemic. While this has been discussed for a number of conditions in the adult population, related patterns have been studied less for children. In light of the detrimental effects of the pandemic, particularly for children and young people under the age of 18, it is pivotal to explore this issue further.

**Methods:**

Based on complete national hospital discharge data available via the German National Institute for the Reimbursement of Hospitals (InEK) data browser, we compare the top 30 diagnoses for which children were hospitalised in 2019, 2020, 2021 and 2022. We analyse the development of monthly admissions between January 2019 and December 2022 for three tracers of variable time-sensitivity: acute lymphoblastic leukaemia (ALL), appendicitis/appendectomy and tonsillectomy/adenoidectomy.

**Results:**

Compared to 2019, total admissions were approximately 20% lower in 2020 and 2021, and 13% lower in 2022. The composition of the most frequent principal diagnoses remained similar across years, although changes in rank were observed. Decreases were observed in 2020 for respiratory and gastrointestinal infections, with cases increasing again in 2021. The number of ALL admissions showed an upward trend and a periodicity prima vista unrelated to pandemic factors. Appendicitis admissions decreased by about 9% in 2020 and a further 8% in 2021 and 4% in 2022, while tonsillectomies/adenoidectomies decreased by more than 40% in 2020 and a further 32% in 2021 before increasing in 2022; for these tracers, monthly changes are in line with pandemic waves.

**Conclusions:**

Hospital care for critical and urgent conditions among patients under the age of 18 was largely upheld in Germany during the COVID-19 pandemic, potentially at the expense of elective treatments. There is an alignment between observed variations in hospitalisations and pandemic mitigation measures, possibly also reflecting changes in demand. This study highlights the need for comprehensive, intersectoral data that would be necessary to better understand changing demand, unmet need/foregone care and shifts from inpatient to outpatient care, as well as their link to patient outcomes and health care efficiency.

**Supplementary Information:**

The online version contains supplementary material available at 10.1186/s12913-023-09929-z.

## Introduction

The COVID-19 pandemic has caused substantial disruptions in the delivery of health services across all service areas and delivery platforms around the world [1]. Drops in physician contacts and non-COVID-19 hospital admissions have been reported for several conditions and a range of countries [2–4].

These disruptions were likely influenced by a range of both supply and demand-side factors. The high numbers of COVID-19 patients put unprecedented pressure on health systems. As a result, a range of measures were implemented to ensure adequate capacities for COVID-19 care and minimise exposure of those seeking care for other reasons [5]. Routine visits and elective procedures were often postponed or cancelled on all levels of care, and post-surgery and palliative care services were discontinued [5]. At the same time, people may have refrained from or delayed seeking in-person medical support to avoid exposure to SARS-CoV-2. Contractions in emergency department admissions for life-threatening conditions, such as stroke and myocardial infarction, have been reported and could impact health outcomes [2, 4].

The majority of research on the disruption of health care services during the COVID-19 pandemic has focused on the adult population [2–4]; less attention seems to have been paid to paediatric care. However, there is evidence from different countries on delayed presentations to paediatric assessment units and general paediatric care [6, 7], reductions in hospital admissions [8] and emergency department visits [9], declining immunisation rates [10], as well as an increase in severe cases of certain conditions such as new onset diabetic ketoacidosis for type 1 diabetes [11]. These patterns are also reflected in findings from Germany for the first wave of the pandemic [12].

The above observations have been raising concerns about access to appropriate and timely care for children during the COVID-19 pandemic and in its aftermath. For some of the observed phenomena, it remains unclear whether they reflect a risk for patients due to unmet care needs, a correction of previous overprovision or inefficient provision of services, or both. Against the backdrop of the detrimental effects of the COVID-19 pandemic and its implications, particularly on those under the age of 18 [13], it is pivotal to explore this issue further.

This study aims to compare hospitalisations for children before and during the COVID-19 pandemic using complete national data from German hospitals and explore potential differences in patterns for conditions and interventions with different levels of urgency and severity. It investigates the top 30 diagnoses for which children were admitted to German hospitals in 2019, 2020, 2021 and 2022 and then zooms into three different tracers (acute lymphoblastic leukaemia, ALL; appendicitis; tonsillectomy/ adenoidectomy) to provide preliminary insights on the relationship between changes in admission rates and time-sensitivity of care.

Germany is a federal republic; lockdown measures during the COVID-19 pandemic were mandated by the national government but implemented at the federal state level, resulting in regional and local variation. The first national lockdown was implemented in mid-March 2020, with social distancing measures persisting at least until early June 2020, and longer in some federal states. The second national lockdown was preceded by a “light” lockdown which came into force on 2 November 2020. Restrictions were hardened in mid-December 2020 and lasted until March 2021. This was followed by revised legislation on health protection that allowed for further flexibility in implementing public health and social measures across the federal states as of April 2021 [14, 15]. During lockdowns, hospitals were advised to postpone elective non-urgent treatments to preserve capacity for the treatment of patients with COVID-19 [5, 16, 17]. Most federal-level measures were lifted following legislative changes in March 2022; the new legal framework also defined narrower scopes for state-level measures, focusing on the protection of vulnerable groups [18].

## Methods

### Data

We used complete national hospital discharge data collected according to § 21 of the Hospital Remuneration Act (*Krankenhausentgeltgesetz*) [19] (the so-called “DRG data”) available through the open access data browser of the German National Institute for the Reimbursement of Hospitals (*Institut für das Entgeltsystem im Krankenhaus*, InEK), to which all German hospitals routinely report performance and billing data (see Sect. 1 of the online Appendix). The data browser was created in response to the COVID-19 pandemic and provides access to aggregated discharge data on all inpatient cases discharged from hospitals in 2019, 2020, 2021 and 2022 including inter alia information on principal and secondary diagnoses (coded according to ICD-10, German modification), performed procedures (coded according to the German Operation and Procedure Classification “OPS”), as well as limited sociodemographic indicators (age group, sex) and other relevant clinical information, such as average length of hospital stay (ALOS) and distribution of hospitalisations by hospital bed capacity [19]. The data captures cases with inpatient stays but not those who were treated as outpatients (e.g., for emergency care or day surgery).

### Data extraction

We obtained data for children aged 28 days to 17 years who were admitted to German hospitals between 1 January 2019 and 31 December 2022 via the InEK data browser interface (https://datenbrowser.inek.org/). We first extracted data on the total number of cases admitted between 1 January and 31 December each year to identify overall changes in volume and the breakdown of annual cases by principal diagnosis. From among the 30 most frequent diagnoses represented each year, we selected three tracers with varying degrees of time-sensitivity: (a) ALL without stated remission (acute essential care [20]); (b) appendicitis (acute care with a potential alternative to immediate hospitalisation [21]); and (c) tonsillectomy/adenoidectomy (planned care to address chronic conditions [22]). These tracers were selected precisely because they could be considered to adequately reflect these different categories of time-sensitivity; this was not applicable for most of the other frequent diagnoses identified in the previous step. We extracted monthly admissions for (a) cases with ALL not having achieved remission (identified via ICD-10 principal diagnosis code C91.00); (b) cases with appendicitis (see Table 1 for ICD-10 principal diagnosis codes) and cases with appendicitis who underwent an appendectomy (identified via the same ICD-10 principal diagnoses plus relevant OPS procedure codes); and (c) cases who underwent a tonsillectomy and/or adenoidectomy (identified via OPS procedure codes, see Table 1). First data samples to conceptualise the study were downloaded in April 2021; data for the final analysis were extracted between 15 and 28 February 2022 for 2019 and 2020 and between 18 and 20 May 2023 for 2021 and 2022.

### Data analysis

Data was analysed using Microsoft Excel. Annual total case numbers for 2019, 2020, 2021 and 2022 were compared and differences were calculated (absolute numbers and percent changes). The 30 principal diagnoses (three-digit ICD-10 categories) with the highest number of cases were listed for each year, and differences to previous years were calculated (absolute numbers and percent changes). For the three tracers (ALL, appendicitis/appendectomy, and tonsillectomy/adenoidectomy), annual and monthly data across the three years were compared and plotted graphically. Differences in sex, age group, ALOS, patient clinical complexity level (PCCL; a measure of the cumulative effect of a patient’s complications and comorbidities calculated for each episode of care), and hospital size were explored descriptively.

## Results

### Overall case numbers and most frequent reasons for hospitalisation

**Fig. 1:**
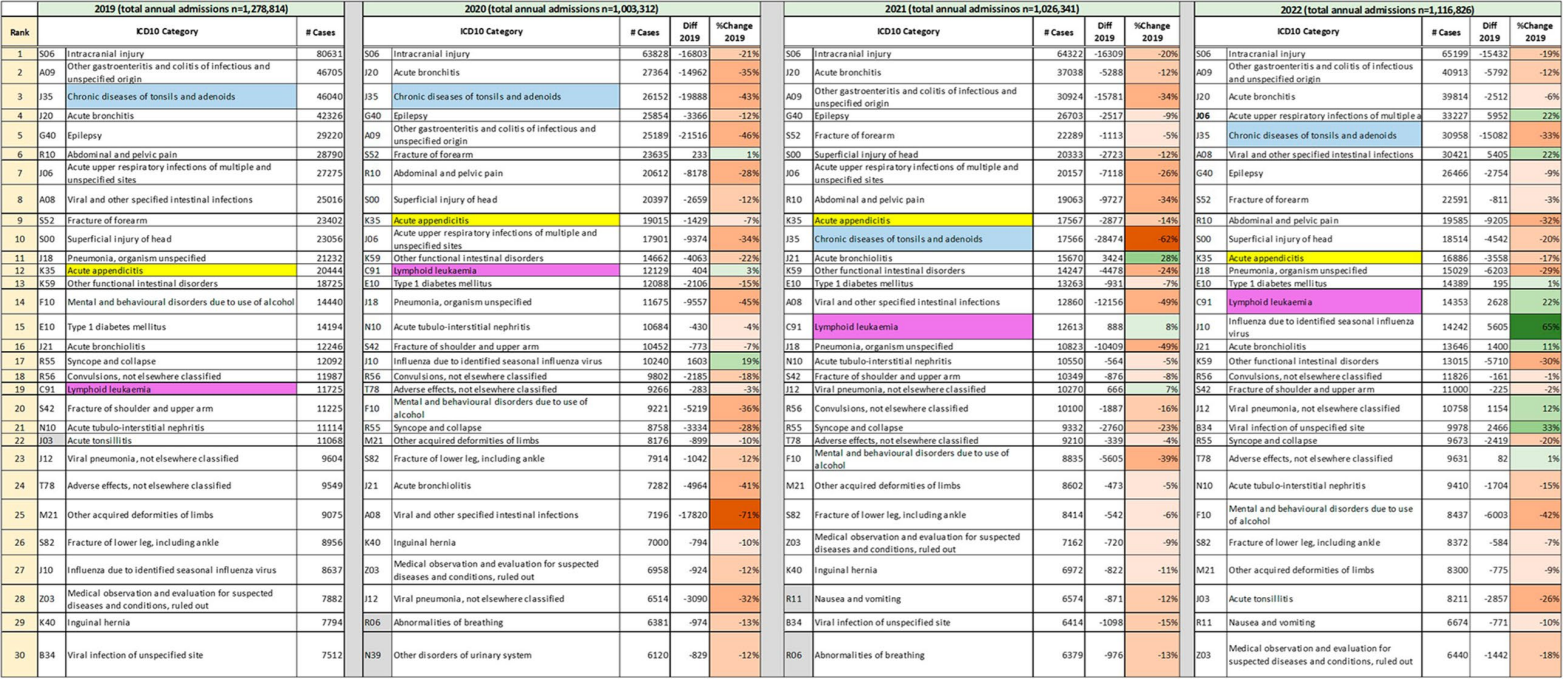
Thirty most frequent discharge diagnoses for hospitalised children in 2019, 2020, 2021 and 2022 Legend: Shading of percent change numbers is based on the following increments from lighter to darker: 0–10%, 11–25%, 26–50%, > 50%. The three tracers analysed in detail are highlighted in different colours (blue, yellow, purple); numbers in the table reflect cases for the three-digit ICD-10 categories and thus do not exactly match data in the detailed analysis. Greyed cells denote conditions not appearing in previous/subsequent years

The total number of admissions decreased by more than 20% in 2020 compared to 2019 (see Fig. 1). In 2021, admissions increased by 2.7% compared to 2020 but remained 19.5% lower than in 2019. In 2022, admissions increased by 8.8% compared to 2021, and stayed 12.7% below 2019 levels. Annual ALOS increased by 0.1 days in 2020 and then remained stable before returning to 2019 levels in 2022 (in days, 2019: 3.2; 2020: 3.3; 2021: 3.3; 2022: 3.2). No obvious patterns emerged in the composition of annual admitted cases regarding sex, age group or patient complexity (see Sect. 2 of the online Appendix).

Figure 1 shows the principal diagnoses for which children received inpatient care in 2019, 2020, 2021 and 2022. Relative changes compared to 2019 are colour-coded to highlight decreases (red) or increases (green). The overall composition of the 30 most frequent diagnoses remained similar across years, but changes in rank can be observed.

For admissions due to respiratory (e.g., J20, J06, J18, J21) and gastrointestinal infections (e.g., A09, A08), decreases between 30% and 70% were observed in 2020 compared to 2019. For some of these conditions, admissions in 2021 remained lower than 2019, but increased compared to 2020 (J20, J06, A09, A08), while for others, admissions in 2021 surpassed 2019 values (e.g., J12 by 7% and J21 by 28%). Admissions for all respiratory and gastrointestinal infections were higher in 2022 compared to 2021, except for acute bronchiolitis (J21); for some conditions (e.g., J06 and A08), admissions in 2022 were more than 20% higher than in 2019. Hospitalised influenza cases increased by nearly 20% in 2020 compared to 2019, and almost disappeared in 2021 (there were 172 hospitalised cases with an ICD-10 code J10 in 2021, a 98% reduction compared to the two previous years); admissions increased again in 2022, surpassing the 2019 value by 65%.

Among hospitalisations for the most frequent non-communicable conditions or clinical signs, decreases of over 20% compared to 2019 were observed in 2020 for chronic diseases of the tonsils and adenoids (43%, K35), mental and behavioural disorders due to use of alcohol (36%, F10), abdominal and pelvic pain (28%, R10), syncope and collapse (28%, R55) and certain functional gastrointestinal disorders (22%, K59). In 2021, admissions for most of these conditions decreased by less than 10% or not at all compared to 2020 (thus remaining at levels at least 20% lower than 2019), with the exception of admissions for chronic diseases of the tonsils and adenoids, which decreased by another 33%. In 2022, admissions for conditions of the tonsils and adenoids increased compared to 2021 and 2020 but remained 33% below 2019 values; for the other four conditions, admissions either increased slightly compared to 2021 (R10, R55) or decreased further (F10, K59), all remaining at least 20% below 2019 levels. For some serious acute conditions, such as acute tubulo-interstitial nephritis (N10), the number of cases decreased by less than 5% in 2020, and kept decreasing over the observation period (2022 admissions 15% below 2019); for others, such as ALL (C91), admissions initially increased by less than 5% and kept increasing over the observation period (2022 admissions 22% above 2019). Admissions due to diabetes mellitus (E10) decreased by 15% in 2020 compared to 2019, and then gradually returned to 2019 levels by 2022.

Injuries of the head and extremities remained among the most frequent reasons for hospitalisation during the observation period (see Fig. 1). Admissions due to intracranial injuries and superficial injuries of the head decreased by 21% and 12% respectively in 2020 compared to 2019 and remained stable in 2021. In 2022, admissions due to intracranial injuries remained 19% lower than 2019, while admissions due to superficial head injuries decreased further to 20% below 2019 levels.

**Table 1:** Characteristics of inpatient cases with acute lymphoblastic leukaemia, appendicitis/appendectomy, and tonsillectomy/adenoidectomy

	2019	2020	2021	2022
**Acute lymphoblastic leukaemia** ^**1**^				
Hospital admissions, n	10,119	10,246	10,661	12,369
Age, %				
28 d – 1 yo^2^ 1–2 yo 3–5 yo 6–9 yo 10–15 yo 16–17 yo	1.62 14.58 33.14 20.87 24.88 4.91	0.61 16.28 35.83 20.05 21.16 6.07	1.81 17.91 32.49 20.34 21.53 5.93	0.70 19.44 32.25 20.62 21.76 5.24
Sex, %				
Male Female	56.61 43.39	58.65 41.35	57.59 42.41	57.26 42.74
PCCL, %^3^				
0 1–6	60.28 39.72	57.36 42.64	58.84 41.16	65.75 34.25
ALOS, mean ± SD	5.5 ± 9.7	5.6 ± 8.9	5.5 ± 8.8	5.7 ± 11.1
**Appendicitis (underwent appendectomy)** ^**4**^				
Hospital admissions, n	22,454 (20,885)	20,363 (19,040)	18,771 (17,384)	18,086 (16,555)
Age, %				
28 d – 1 yo 1–2 yo 3–5 yo 6–9 yo 10–15 yo 16–17 yo	0.01 (0.01) 0.90 (0.72) 4.66 (4.39) 17.59 (17.35) 53.18 (53.19) 23.67 (24.34)	0.04 (0.03) 0.91 (0.74) 5.03 (4.78) 19.31 (19.02) 52.22 (52.30) 22.49 (23.13)	0.08 (0.07) 0.86 (0.70) 5.72 (5.44) 19.80 (19.66) 50.99 (50.95) 22.55 (23.17)	0.06 (0.05) 0.78 (0.69) 5.52 (5.19) 20.25 (19.91) 50.82 (50.66) 22.56 (23.49)
Sex, %				
Male Female	50.90 (50.98) 49.09 (49.01)	52.33 (52.44) 47.66 (47.55)	53.01 (53.26) 46.99 (46.73)	53.88 (54.07) 46.10 (45.90)
Principal diagnosis, %				
K35.30, K35.8, K36, K37 - uncomplicated appendicitis K35.2, K35.31, K35.32 - complicated appendicitis	82.53 (82.52) 17.47 (17.48)	80.40 (80.06) 19.60 (19.94)	79.84 (79.47) 20.16 (20.53)	78.92 (78.34) 21.08 (21.66)
PCCL, %				
0 1–6	83.99 (83.61) 16.01 (16.39)	82.33 (81.99) 17.67 (18.03)	81.09 (80.64) 18.91 (19.37)	79.09 (78.44) 20.89 (21.57)
ALOS, mean ± SD	4.3 ± 2.8 (4.3 ± 2.7)	4.3 ± 2.9 (4.3 ± 2.8)	4.2 ± 2.8 (4.2 ± 2.7)	4.2 ± 2.9 (4.2 ± 2.8)
**Tonsillectomy and/or Adenoidectomy** ^**5**^				
Hospital admissions, n	51,370	29,148	19,864	34,174
Age, %				
28 d – 1 yo^4^ 1–2 yo 3–5 yo 6–9 yo 10–15 yo 16–17 yo	0.65 19.51 46.97 16.42 10.71 5.74	0.82 18.71 47.49 16.07 10.49 6.42	1.27 22.97 45.86 12.03 10.01 7.85	0.72 19.46 53.90 13.22 7.15 5.56
Sex, %				
Male Female	56.02 43.97	56.52 43.47	55.96 44.00	57.64 42.32
Principal diagnosis, %				
J35.2 (hypertrophy of adenoids) J35.3 (hypertrophy of tonsils & adenoids) J35.0 (chronic tonsillitis & adenoiditis) J35.1 (hypertrophy of tonsils) Other principal diagnosis	29.48 28.68 17.26 11.99 12.52	26.04 29.54 17.61 13.48 13.33	27.76 27.25 16.35 13.89 13.27	31.95 30.22 12.52 12.65 11.60
PCCL, %^3^				
0 1–6	93.87 6.13	93.66 6.34	92.43 7.57	93.02 6.98
ALOS, mean ± SD	2.5 ± 2.2	2.5 ± 2.2	2.4 ± 2.1	2.2 ± 2.0

^1^ Cases with a principal diagnosis of C91.00 (ICD-10)

^2^ yo = years of age

^3^ PCCL = Patient Clinical Complexity Level; levels 0 (no comorbidities/complications), 1 (light comorbidities/complications) to 6 (most severe comorbidities/complications)

^4^Cases with a principal diagnosis in K35, K36, K37 (ICD-10); cases who underwent appendectomy (principal diagnosis in K35, K36, K37 and procedure code in 5-470, 5-455.3) are shown in brackets

^5^Cases with a procedure code in 5-281, 5-282, 5-285 (OPS)

### Acute lymphoblastic leukaemia (ALL)

**Fig. 2:**
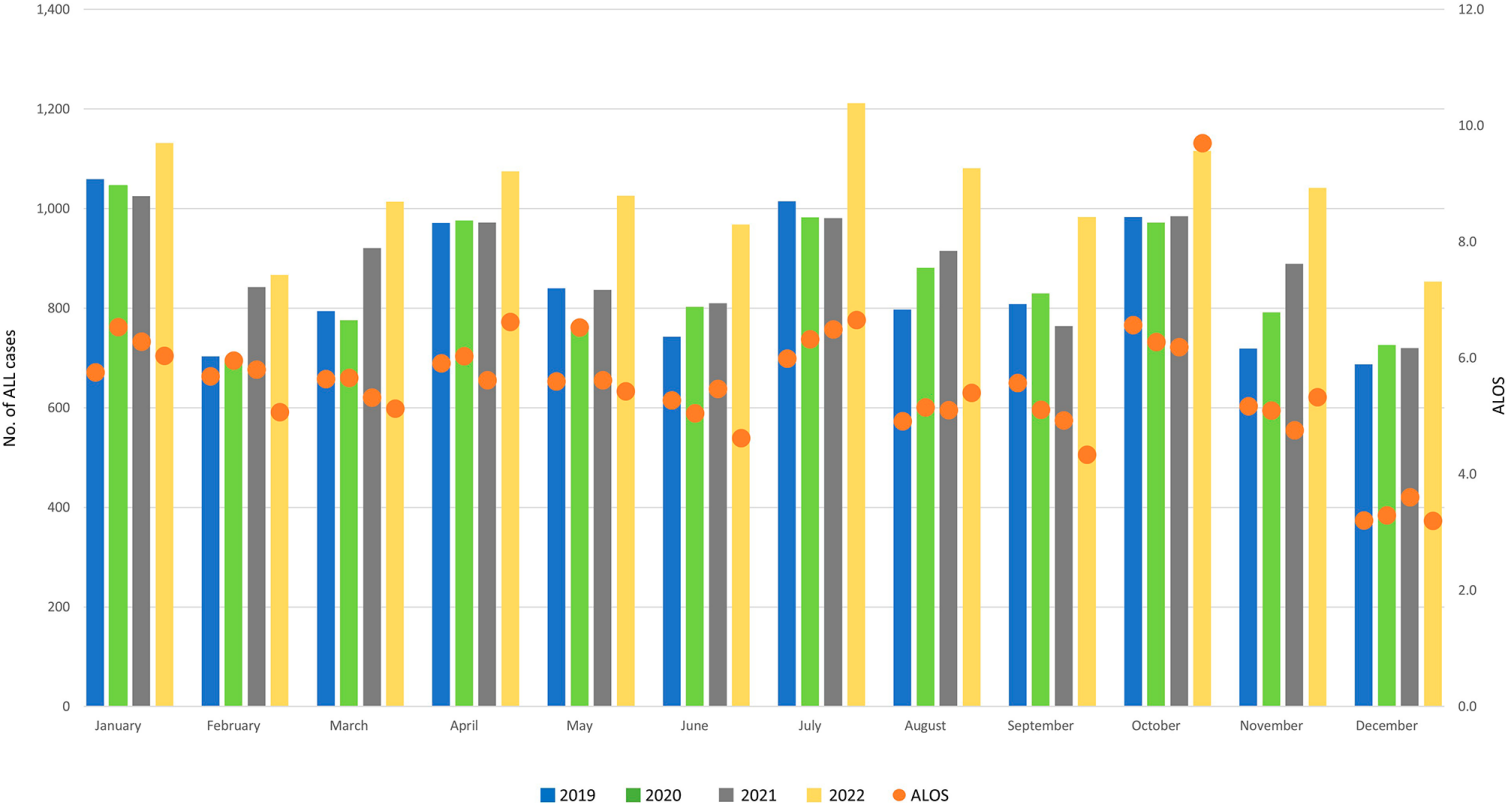
Monthly hospital admissions and ALOS for ALL, January 2019 – December 2022

The total number of children hospitalised for ALL not having achieved remission (C91.00) increased by 1.3% from 2019 to 2020, by 4.1% from 2020 to 2021, and by 13.8% from 2021 to 2022 (see Table 1). Monthly case numbers show that fluctuations throughout the year followed four “peaks” across the observation period, in January, April, July, and October of each year (see Fig. 2 and Figure S1 in the online Appendix for relative changes). The composition of ALL cases regarding sex, age group and patient complexity shows variations throughout the observation period (see Table 1 and Sect. 3 of the online Appendix). ALOS ranges from 3.2 days (December 2019 and December 2022) to 9.7 days (October 2022), with a median of 5.6 days. The share of cases treated in large tertiary hospitals of 1000 beds or more decreased by approximately 10% points in 2020 (see online Appendix), while the share of ALL cases admitted in hospitals of under 1000 beds increased; admissions in hospitals with 1000 beds or more increased again in 2021 and 2022.

### Appendicitis and appendectomy

**Fig. 3:**
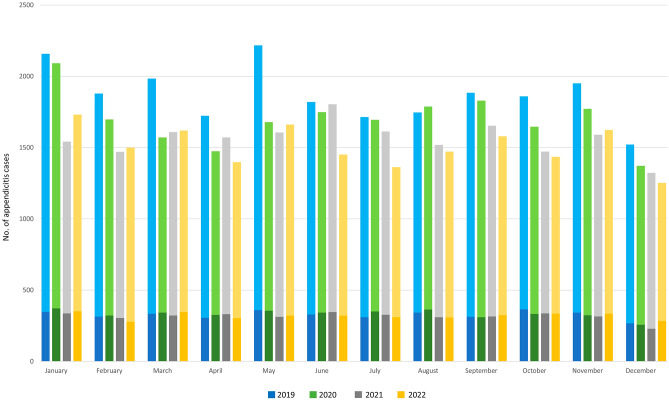
Monthly hospital admissions for appendicitis by level of complication, January 2019 - December 2022. *Note*: darker column segments correspond to complicated cases, lighter segments to uncomplicated cases

Hospital admissions for children diagnosed with appendicitis decreased by 9.3% between 2019 and 2020, by 7.8% between 2020 and 2021 and by 3.7% between 2021 and 2022 (see Table 1). Total monthly admissions due to appendicitis were below 2019 levels for the duration of the observation period with the exception of August 2020 (see Fig. 3 and Sect. 4 of the online Appendix).

There were more male patients hospitalised for appendicitis than females across all years; the proportion of male cases increased over the observation period (Table 1). Across years, most admissions regarded patients aged 10 to 15 years. Among cases admitted for appendicitis, the vast majority underwent appendectomy (2019: 93.0%, 2020: 93.5%, 2021: 92.4%, 2022: 91.5%).

The majority of hospitalised appendicitis cases had an uncomplicated clinical presentation based on ICD-10 diagnosis [23] (see Figs. 3 and 2019: 82.5%, 2020: 80.4%, 2021: 79.8%, 2022: 78.92%). The share of cases with complications such as generalised peritonitis, abscess formation, rupture or perforation increased across years (2019: 17.5%, 2020: 19.6%, 2021: 20.2%, 2022: 21.1%). The highest relative reductions (≥ 20%) in admissions for uncomplicated appendicitis compared to 2019 were observed in March and May 2020, in January to March, May as well as October and November 2021 and for most months in 2022, with the exception of August and November; relative increases in complicated cases were observed for some months over the observation period, but they only surpassed 10% of the 2019 value in July 2020 (see Figure S5 in the online appendix).

The share of cases with low clinical complexity (PCCL level 0) decreased between 2019 and 2022, while the share of cases with higher complexity rose from 16.0 to 20.9% in all cases and from 16.4 to 21.6% in cases with appendectomy, respectively. ALOS remained similar across years (see Table 1).

Across all years, small- to medium-sized hospitals (200–599 beds) hosted most cases, followed by large centres of 1000 beds and more. Over this period, reductions in admissions occurred in hospitals with fewer than 400 beds (see online Appendix).

### Tonsillectomy and adenoidectomy

**Fig. 4:**
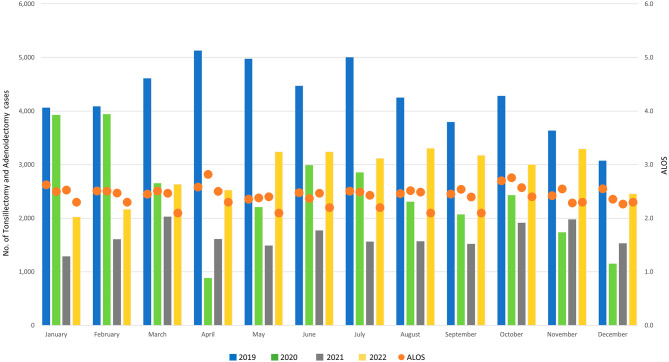
Monthly hospital admissions for tonsillectomy/adenoidectomy, January 2019 - December 2022

The number of cases hospitalised for tonsillectomy and/or adenoidectomy was 43.2% lower in 2020 compared to 2019, and decreased by a further 31.7% in 2021; in 2022, cases increased by 72% compared to 2021, but remained 33.5% below 2019 levels (see Table 1). The largest monthly reductions in 2020 compared to the corresponding months in 2019 were observed in April (82.8%) and May (55.6%), as well as November (52.2%) and December (62.6%) (see Fig. 4 and Figure S8 in the online appendix). Admissions for all months in 2021 remained over 50% below 2019 levels, with the exception of November (-45.7%); they were also lower than the corresponding 2020 values for most months, except April (+ 83.3%), November (+ 13.8%) and December (+ 33.0%). In 2022, monthly admissions surpassed those in 2021 throughout the year; they remained more than 40% below 2019 until April, but relative differences diminished to below 40% from May onwards (see Figure S8 in the online appendix).

The age group with the highest share of tonsillectomies and/or adenoidectomies was that of children aged 3 to 5 years; small variations in the shares of the remaining age groups can be observed throughout the observation period.

In all three years, the most frequent underlying diagnoses for tonsillectomies and/or adenoidectomies were hypertrophy of the adenoids (ICD code J35.2), hypertrophy of the tonsils with hypertrophy of adenoids (J35.3), chronic tonsillitis and adenoiditis (J35.0) and hypertrophy of the tonsils only (J35.1), adding up to over 85% of diagnoses for all cases (see Table 1).

The share of cases with a PCCL of level 1 and above increased from 6.1% to 2019 to 7.5% in 2021, and decreased to 6.98% in 2022. ALOS remained stable in 2020, but decreased by 0.1 days in 2021 and a further 0.2 days in 2022 (see Table 1).

The distribution of tonsillectomies and adenoidectomies across hospitals of different sizes remained largely the same during the observation period; the proportion of cases treated in the smallest hospitals (less than 200 beds) increased by nearly 3% points (9.9–12.7%) from 2019 to 2021 before declining in 2022 (see Sect. 5 of the online Appendix).

### Comparison of changes for ALL, appendicitis, and tonsillectomies/adenoidectomies

**Fig. 5:**
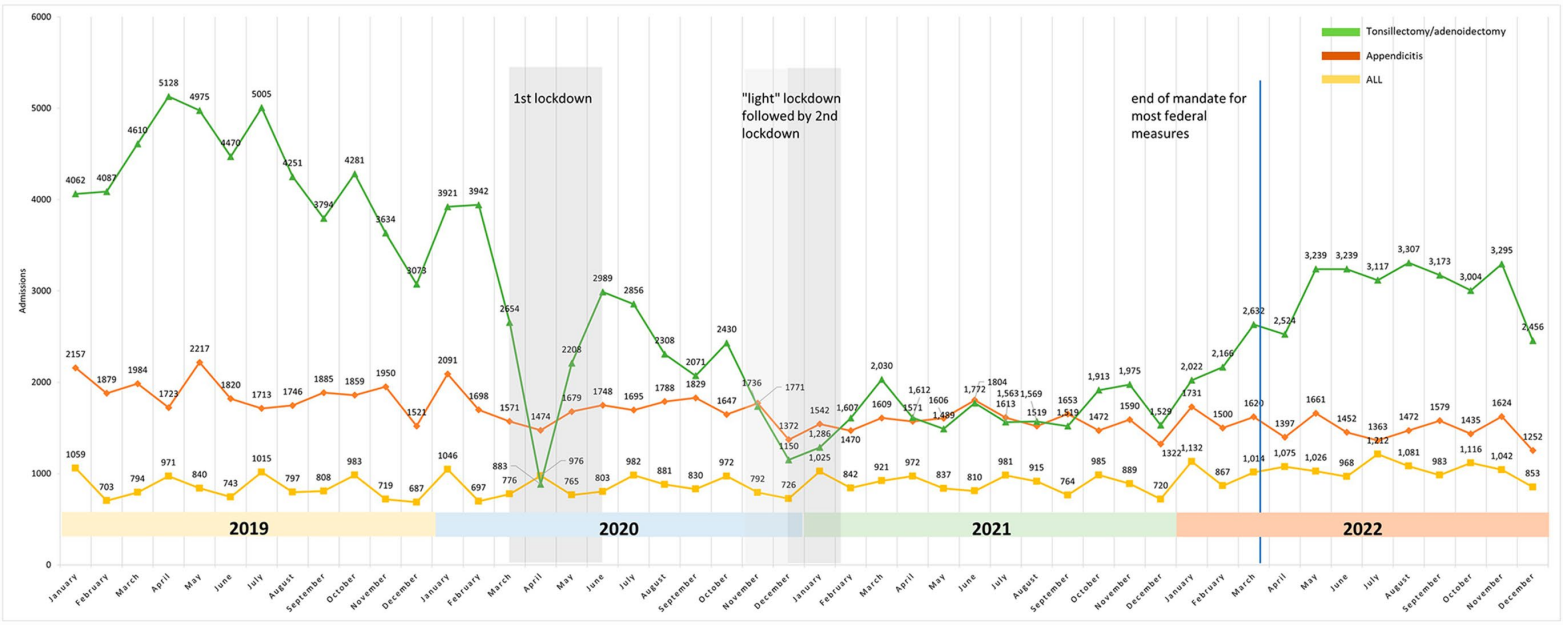
Monthly case numbers for ALL, appendicitis and tonsillectomy/adenoidectomy between January 2019 and December 2022 and federal lockdown measures in Germany (state level measures are not depicted)

Figure 5 plots the monthly admission numbers for the three tracers against lockdown periods in Germany. It shows that these periods coincided with decreases in admissions for tonsillectomies/adenoidectomies and appendicitis, but not for ALL. Following the end of the federal mandate for most pandemic-related restrictions in March 2022, an increase in monthly admission numbers for tonsillectomy/adenoidectomy can be observed; no similar effect is obvious for appendicitis or ALL.

## Discussion

The number of paediatric hospitalisations in Germany fell by 20% in 2020 compared to 2019. Although admissions increased again slightly in 2021, and again more substantially in 2022, the number of inpatient cases did not return to pre-pandemic levels by the end of the observation period. Similar findings have been reported in adult populations, with persistent reductions in both elective and emergency routine hospital care documented in Germany and worldwide [24, 25]. Previous work on a sample of German paediatric patients showed a drop of around 40% for overall admissions and surgeries in the first months of the pandemic [12].

Overall, the composition of the most frequent clinical indications for admission did not change substantially. However, individual diagnoses displayed important fluctuations throughout the pandemic. In particular, respiratory and gastrointestinal infections plummeted in 2020, likely as a result of COVID-19 restrictions; this confirms previous findings from the German and international literature [26, 27]. Remarkably, influenza cases displayed an initial soar in 2020, as many children were likely admitted on suspicion of a SARS-CoV-2 infection, but subsequently reached a record low in 2021 [26]. Influenza admissions rose again dramatically in 2022, potentially reflecting increased susceptibility following the period of non-exposure due to pandemic mitigation measures [28] in combination with increased testing activity [29]. The same interpretation could explain increases in other infectious diseases observed in 2021 and 2022 compared to 2019. For instance, the considerable rise in admissions for acute bronchiolitis in 2021, which was largely driven by an increase in respiratory syncytial virus (RSV) infections (ICD-10 code J21.5, effect masked in Table 1 due to the aggregation of cases to three-digit ICD-10 categories), has also been observed elsewhere and linked to the restrictions implemented to halt viral transmission [30]. The early start of the influenza season in 2022, combined with increased hospitalisations in children due to RSV and persistent COVID-19 concerns put European health systems under duress in winter 2022 [31].

Changes in admissions for non-communicable conditions and injuries in 2020 and 2021 could reflect behavioural changes produced by restrictions such as the closure of schools and reduced social interactions with other children [12], or changes in clinical practice in light of pandemic mitigation measures. For example, admissions due to diabetes mellitus decreased in 2020, and picked up again in 2021, finally reaching 2019 levels in 2022. For indications with decreases in admissions that matched or surpassed 2021 levels in 2022 (a year with fewer pandemic-related restrictions), such as head injuries and functional gastrointestinal disorders, further analyses should explore the potential link with an evolution of clinical protocols. Admissions to German hospitals due to behavioural and mental disorders triggered by alcohol consumption decreased continuously over the observation period, but it remains unclear whether this is related to a decrease in alcohol consumption or a rise in unmet care needs.

Hospitalisation rates for the three tracers were affected to varying degrees throughout the observation period. The lack of substantial shifts in ALL hospital admissions suggests that inpatient services for severe conditions, such as haematologic cancers, were upheld throughout the pandemic. The increase in active ALL admissions over the observation period is in line with data from the German Childhood Cancer Registry [32], and merits further investigation. Despite this increase in cases, the share of ALL patients treated in large tertiary centres decreased during the pandemic in favour of smaller hospitals, which may reflect the reallocation of patients and resources implemented as part of the pandemic response. The four peaks in the distribution of hospitalisations for ALL consistently observed for every year of the observation period correspond to the first month of every quarter and could be related to therapeutic regime planning or other contextual factors.

Observed reductions in appendicitis hospitalisations in 2020 and 2021 were most pronounced for the months overlapping with the pandemic mitigation measures implemented by the government and federal states; however, admissions remained at least 15% below 2019 levels for every month in 2022, which might reflect an evolution in clinical practice towards outpatient management [33]. There was a slight increase in the proportion of complicated clinical presentations over the observation period, with similar findings reported in the literature [34]. However, without comparable data from ambulatory and emergency department settings, it is impossible to judge whether this was due to fear of exposure to SARS-CoV-2 while seeking care and related delays, or other factors [35].

The observed reductions in tonsillectomy and/or adenoidectomy procedures reflect the widespread postponement of elective interventions supported by the German government in mid-March 2020, while changes in demand due to concerns about SARS-CoV-2 exposure may also have played a role. These findings are in line with a population-wide analysis across all age groups, which also highlights the evolution of clinical practice towards minimally invasive approaches performed in ambulatory settings [17]. While it is conceivable that the pandemic further accelerated this development, the renewed increase in admissions following the lapse of federal restrictions in March 2022 observed in our data might suggest otherwise and requires further investigation.

In general, shifts from inpatient to outpatient treatment could not be investigated in this study due to the nature of the data. However, the plausibility of ambulatory care fully offsetting the observed decreases in inpatient care is questionable, given that outpatient care visits in Germany also decreased markedly during lockdown periods, particularly for children, and did not increase substantially overall during the pandemic. A similar pattern can be observed for outpatient surgery [36].

Our study has several limitations. Given the research question(s), this was by definition a retrospective design, and draws on aggregated data based on ICD-10 codes used for billing purposes. On the one hand, there is a risk of misdiagnosis that is impossible to account for in such a design; what is more, admissions motivated by social circumstances rather than the severity of presentation cannot be discerned, although this distinction would have been particularly meaningful to capture in an analysis like this one. Furthermore, hospital discharge data are typically prone to entry and codification errors; however, German hospital billing data are considered fairly reliable with regard to reimbursement-relevant content [37]. The DRG data capture all national inpatient cases, with the exception of cases financed through the statutory insurance for occupational accidents and treated in occupational health hospitals; as they represent only small case numbers, the DRG data can be assumed to be virtually complete. Linkage to outpatient data would require a more complex methodological approach than the one adopted here; such analyses could draw on the findings of this exploratory descriptive study (see below). Since the InEK data browser only contains pre-pandemic data for 2019, it was impossible to account for particularities in hospital admission numbers of that year or compare hospitalisation trends over several years, including decreasing tendencies in admissions for appendicitis and/or tonsillectomies/adenoidectomies before the pandemic. Additionally, the dataset used for 2022 was still subject to updates and corrections by submitting hospitals at the time of writing; this might have biased the results, but potential changes are not expected to be of a magnitude that would substantially change the overall interpretation of our findings. The data browser only provides a limited set of sociodemographic indicators, only for inpatient cases (not patients) and at an aggregate level; this precludes stratification by specific patient subgroups as well as following up on individual patients to identify those with multiple hospitalisations. Thus, the data available through the data browser does not lend itself to further inferential analyses, which were therefore not attempted here. Given the nature of the data, this work was conceived as an exploratory descriptive analysis to identify patterns and generate hypotheses; as such, we did not test differences between years for statistical significance. Finally, the three tracers are not representative of all urgent or elective care in the German health system, and our analysis of the most frequent 30 diagnoses remained superficial, but can provide impetus for further investigations.

Indeed, follow-up analyses could further explore changes in the most frequent reasons for admission for narrower age groups or focus on case distribution by hospital size for specific indications or indication groups. What is more, future research should go beyond pattern description to understand if observed differences in admission rates have led to adverse outcomes for paediatric patients during the pandemic; this would require adequate data. For the German context, this might entail working with individual sickness funds to be able to depict the entire patient pathway. Understanding the determinants of observed hospitalisation patterns requires primary research, especially since the decision to admit a child can sometimes be motivated by social circumstances rather than severity of clinical presentation. International evidence indicates inequalities in healthcare disruptions during the pandemic, both for the general population [38] and for paediatric care [39]; this dimension would also be crucial to investigate further in the German setting.

## Conclusions

This is the first nationally complete study capturing all paediatric inpatient cases in Germany in the immediate pre-pandemic and pandemic periods. Our study demonstrates that paediatric care for critical and urgent conditions was largely upheld, potentially at the expense of elective treatments. This must be interpreted in the context of Germany’s health system, which has a large inpatient sector and relative overcapacity in terms of hospital beds [40]; it is likely that the situation has been quite different in other settings. Indeed, there are growing concerns over looming backlogs of health services, which threaten to put additional strain on health systems globally [41]. As policies to address this issue are developed, particularly understudied and vulnerable groups like children should stay in focus. This study also demonstrates the need for comprehensive, intersectoral data that enable a better understanding of changing demand, unmet need, and foregone care as well as shifts from inpatient to outpatient care, and their link to patient outcomes and health care efficiency.

### Electronic supplementary material

Below is the link to the electronic supplementary material.

Supplementary Material 1

## Data Availability

The data used for the analysis are publicly available and freely downloadable from the German National Institute for the Reimbursement of Hospitals (InEK) data browser. All analyses have been documented using Microsoft Excel and will be made publicly available in an open repository prior to publication.
