# Supplementary material for: How did the COVID-19 pandemic affect inpatient care for children in Germany? An exploratory analysis based on national hospital discharge data
Source: BMC Health Serv Res. 2023 Aug 31;23:938. doi: 10.1186/s12913-023-09929-z (PMC10472716; doi:10.1186/s12913-023-09929-z)

ONLINE APPENDIX

Contents

Section 1: Data characteristics 2

Section 2: Characteristics of total annual admissions 3

Section 3: Acute lymphoblastic leukaemia 4

Section 4: Appendicitis/Appendectomy 10

Section 5: Tonsillectomy/Adenoidectomy 13

Section 1: Data characteristics

Table S1: Data characteristics for years 2019, 2020, 2021 and 2022 according to the manual of the InEK data browser (version 28.03.2023)[1]

**Data for 2019 from "Data delivery DRG 2019 grouped by 2020 guidelines"**	**Data for 2020 from "Data delivery DRG 2020 grouped by 2021 guidelines"**	**Data for 2021 from "Data delivery DRG 2021 grouped by 2022 guidelines"**	**Data for 2022 from "Intra-year data delivery DRG January to December 2021"**
• Data for the billing area "DRG" from the data delivery according to § 21 KHEntgG as of 31.03.2020 for the data year 2019<br>• Data grouped according to aG-DRG version 2019_2020. The grouping according to aG-DRG version 2019_2020 uses the same DRGs as interim data deliveries during the year in accordance with § 24 Para. 2 KHG for 2020 and thus enables a direct comparison.<br>• Principal and secondary diagnoses according to ICD-10-GM Version 2019, procedures according to OPS Version 2019.<br>• All data deliveries of hospitals with at least one treatment case in the billing area "DRG" submitted to the data centre by 31.05.2020 are included in the database.<br>• Data supplied by hospitals; contain service data on all full and partial inpatient treatment cases discharged in the calendar year 2019 with care in main or inpatient departments.<br>• Data has successfully passed the quality check ("error procedure").	• Data for the billing area "DRG" from the data delivery according to § 21 KHEntgG as of 31.03.2021 for the data year 2020<br>• Data grouped according to aG-DRG version 2020_2021. The grouping according to aG-DRG Version 2020_2021 uses the same DRGs as interim data deliveries during the year in accordance with Section 24 (2) KHG for 2021 and thus enables a direct comparison.<br>• Principal and secondary diagnoses according to ICD-10-GM Version 2020, procedures according to OPS Version 2020.<br>• All data deliveries of hospitals with at least one treatment case in the billing area "DRG" submitted to the data centre by 31.05.2021 are included in the database.<br>• Data supplied by the hospitals; contain service data on all full and partial inpatient treatment cases discharged in the calendar year 2020 with care in main or inpatient departments.<br>• Data has successfully passed the quality check ("error procedure").	• Data for the billing area "DRG" from the data delivery according to § 21 Para. 3b KHEntgG as of 31.03.2022 for the data year 2021.<br>• Data grouped according to aG-DRG Version 2021_2022<br>• Principal and secondary diagnoses according to ICD-10-GM version 2021, procedures according to OPS version 2021.<br>• All data deliveries of hospitals with at least one treatment case in the "DRG" billing area submitted to the data centre by 31 May 2022.<br>• Data supplied by hospitals; contain service data on all services provided in the calendar year 2021 for full and partial inpatient treatment cases with care in main or inpatient departments.<br>• Data has successfully passed the quality check ("error procedure").	• Data for the billing area "DRG" from the data delivery according to § 21 Para. 3b KHEntgG as of 16.01.2023 for the data year 2021.<br>• Data grouped according to aG-DRG Version 2022<br>• Principal and secondary diagnoses according to ICD-10-GM version 2022, procedures according to OPS version 2022.<br>• All data deliveries of hospitals with at least one treatment case in the "DRG" billing area submitted to the data centre by 16 January 2023.<br>• Data supplied by hospitals; contain service data on all services provided between 01.01.2022-31.12.2022 for full and partial inpatient treatment cases with care in main or inpatient departments.<br>• Data has successfully passed the quality check ("error procedure").

Notes: DRG Diagnosis Related Groups; KHEntgG Krankenhausentgeltgesetz (Hospital Remuneration Act); KHG: Krankenhausfinanzierungsgesetz (Hospital Funding Act)

[1] https://datenbrowser.inek.org/

Section 2: Characteristics of total annual admissions

Table S2: Characteristics of total annual admissions in German hospitals (children until the age of 18, newborns excluded)

**2019**							
**Cases**		**Length of Stay**		**PCCL**		**Age group**	
Nr. Of cases	1,278,814	Short stays	29.37%	0	83.85%		
		Normal stays	68.51%	1	7.18%	28 days - 1 year	13.94%
**Sex**		Long stays	2.12%	2	4.19%	1-2 years	16.90%
males	53.29%			3	3.47%	3-5 years	15.61%
females	46.71%	ALOS	3.2	4	1.09%	6-9 years	14.27%
other	0.00%	Standard deviation ALOS	5.6	5	0.20%	10-15 years	26.41%
unknown	0.00%			6	0.03%	16-17 years	12.86%
**2020**							
**Cases**		**Length of Stay**		**PCCL**		**Age group**	
Nr. Of cases	1,003,312	Short stays	29.32%	0	84.38%		
		Normal stays	68.38%	1	6.42%	28 days - 1 year	14.29%
**Sex**		Long stays	2.29%	2	4.45%	1-2 years	15.85%
males	53.38%			3	3.43%	3-5 years	15.35%
females	46.62%	ALOS	3.3	4	1.07%	6-9 years	14.59%
other	0.00%	Standard deviation ALOS	5.9	5	0.21%	10-15 years	26.85%
unknown	0.00%			6	0.03%	16-17 years	13.08%
**2021**							
**Cases**		**Length of Stay**		**PCCL**		**Age group**	
Nr. Of cases	1,026,341	Short stays	30.00%	0	83.93%		
		Normal stays	67.81%	1	6.75%	28 days - 1 year	15.08%
**Sex**		Long stays	2.19%	2	4.43%	4.52%	17.13%
males	53.43%			3	3.45%	3-5 years	15.53%
females	46.56%	ALOS	3.3	4	1.11%	6-9 years	13.65%
other	0.00%	Standard deviation ALOS	6.0	5	0.20%	10-15 years	25.79%
unknown	0.01%			6	0.03%	16-17 years	12.82%
**2022**							
**Cases**		**Length of Stay**		**PCCL**		**Age group**	
Nr. Of cases	1,116,826	Short stays	30.61%	0	81.42%		
		Normal stays	67.34%	1	9.01%	28 days - 1 year	14.52%
**Sex**		Long stays	2.05%	2	5.00%	1-2 years	16.97%
males	54.13%			3	3.36%	3-5 years	17.29%
females	45.86%	ALOS	3.2	4	1.01%	6-9 years	14.68%
other	0.00%	Standard deviation ALOS	5.7	5	0.18%	10-15 years	24.77%
unknown	0.01%			6	0.02%	16-17 years	11.76%

Patient Clinical Complexity Level (PCCL) – A quantitative measure of the cumulative effects of patient complications/comorbidities per clinical episode

0 – No complication or comorbidity (CC) effect; 1 – Minor CC effect; 2 – Moderate CC effect; 3 – Severe CC effect; 4 – Extremely severe CC effect; 5/6 – Catastrophic CC effect

Section 3: Acute lymphoblastic leukaemia

Figure S1: % change in monthly hospital admissions and ALOS for ALL in 2020, 2021 and 2022 compared to baseline year 2019

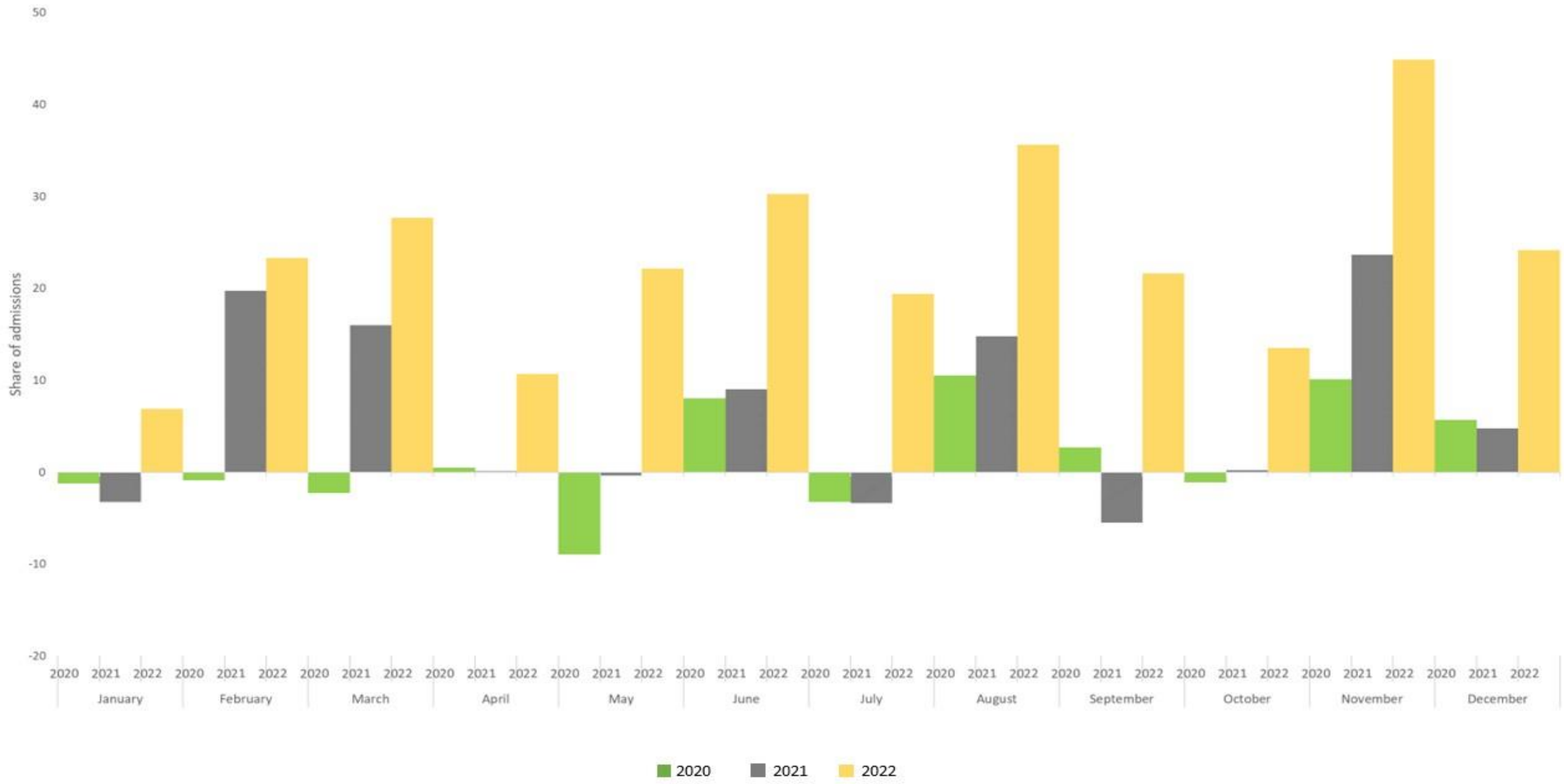

Table S3: Sex and age distribution of admitted ALL patients

		Gender		Age group					
		male	female	28 d - 1 y	1-2 y	3-5 y	6-9 y	10-15 y	16-17 y
**2019**									
Total	10,119	56.61%	43.39%	1.62%	14.58%	33.14%	20.87%	24.88%	4.91%
January	1,059	54.20%	45.80%	1.42%	13.69%	31.82%	20.11%	27.20%	5.76%
February	703	54.91%	45.09%	0.85%	14.94%	34.85%	20.63%	22.90%	5.83%
March	794	54.03%	45.97%	1.26%	15.24%	33.38%	19.40%	25.69%	5.04%
April	971	53.66%	46.34%	1.34%	14.32%	29.87%	22.86%	27.29%	4.33%
May	840	51.67%	48.33%	2.14%	15.36%	35.36%	19.17%	24.05%	3.93%
June	743	56.93%	43.07%	2.02%	15.88%	30.69%	21.94%	24.50%	4.98%
July	1,015	57.44%	42.56%	1.58%	15.96%	29.16%	20.99%	27.49%	4.83%
August	797	60.10%	39.90%	1.00%	15.68%	31.24%	20.95%	26.73%	4.39%
September	808	58.04%	41.96%	2.10%	14.48%	35.77%	20.67%	23.64%	3.34%
October	983	60.83%	39.17%	2.03%	15.06%	34.28%	20.96%	23.09%	4.58%
November	719	57.86%	42.14%	1.81%	12.93%	36.72%	22.11%	19.33%	7.09%
December	687	60.55%	39.45%	1.89%	10.63%	37.26%	20.67%	24.31%	5.24%
**2020**									
Total	10,246	58.65%	41.35%	0.61%	16.28%	35.83%	20.05%	21.16%	6.07%
January	1,046	59.18%	40.82%	0.67%	13.48%	35.18%	21.89%	23.14%	5.64%
February	697	57.53%	42.47%	1.15%	14.63%	34.43%	21.23%	22.38%	6.17%
March	776	56.44%	43.56%	0.39%	17.01%	37.63%	19.85%	18.04%	7.09%
April	976	57.79%	42.21%	1.02%	16.29%	34.53%	20.80%	21.52%	5.84%
May	765	58.56%	41.44%	1.05%	17.12%	36.73%	18.56%	21.18%	5.36%
June	803	55.92%	44.08%	0.37%	18.56%	35.12%	20.17%	20.30%	5.48%
July	982	57.33%	42.67%	0.51%	17.11%	35.95%	17.62%	21.69%	7.13%
August	881	61.98%	38.02%	0.23%	18.73%	35.75%	19.52%	20.09%	5.68%
September	830	61.69%	38.31%	0.36%	16.27%	36.14%	19.28%	20.12%	7.83%
October	972	57.20%	42.80%	0.51%	13.89%	35.39%	22.02%	22.94%	5.25%
November	792	58.84%	41.16%	0.51%	16.67%	34.72%	20.83%	21.72%	5.56%
December	726	61.57%	38.43%	0.69%	16.39%	39.12%	18.18%	19.70%	5.92%
**2021**									
Total	10,661	57.59%	42.41%	1.81%	17.91%	32.49%	20.34%	21.53%	5.93%
January	1,025	60.20%	39.80%	1.27%	14.34%	34.24%	21.66%	22.54%	5.95%
February	842	59.38%	40.62%	2.38%	13.42%	33.97%	21.26%	22.45%	6.53%
March	921	57.55%	42.45%	1.52%	15.96%	34.64%	21.06%	22.48%	4.34%
April	972	55.14%	44.86%	1.13%	14.71%	33.74%	19.44%	24.07%	6.89%
May	837	56.75%	43.25%	1.55%	15.89%	32.86%	21.15%	22.94%	5.62%
June	810	60.12%	39.88%	2.22%	19.51%	32.35%	20.12%	19.88%	5.93%

		Gender		Age group					
		male	female	28 d - 1 y	1-2 y	3-5 y	6-9 y	10-15 y	16-17 y
July	981	59.02%	40.98%	1.94%	19.78%	31.60%	21.00%	21.00%	4.69%
August	915	58.25%	41.75%	1.20%	18.14%	34.54%	20.33%	21.09%	4.70%
September	764	57.72%	42.28%	1.96%	21.73%	30.37%	19.50%	21.86%	4.58%
October	985	57.06%	42.94%	2.34%	21.12%	29.54%	19.90%	19.29%	7.82%
November	889	55.34%	44.66%	2.59%	20.25%	31.38%	18.34%	20.02%	7.42%
December	720	53.89%	46.11%	1.81%	21.39%	29.86%	20.00%	20.42%	6.53%
**2022**									
Total	12,369	57.26%	42.74%	0.70%	19.44%	32.25%	20.62%	21.76%	5.24%
January	1,132	57.24%	42.76%	1.50%	26.50%	26.86%	21.02%	18.46%	5.65%
February	867	52.71%	47.29%	1.27%	27.34%	25.49%	23.99%	17.88%	4.04%
March	1,014	53.55%	46.45%	0.89%	23.57%	29.29%	24.56%	16.77%	4.93%
April	1,075	57.86%	42.14%	0.65%	20.93%	31.26%	22.60%	19.35%	5.21%
May	1,026	54.78%	45.22%	0.97%	21.83%	30.41%	19.79%	23.10%	3.90%
June	968	58.47%	41.53%	0.21%	18.80%	34.50%	18.18%	22.62%	5.68%
July	1,212	59.49%	40.51%	0.17%	16.25%	35.15%	19.39%	23.18%	5.86%
August	1,081	60.41%	39.59%	0.37%	16.56%	34.41%	20.26%	22.48%	5.92%
September	983	59.00%	41.00%	0.61%	14.95%	35.91%	19.53%	23.19%	5.80%
October	1,116	57.97%	42.03%	0.45%	16.04%	34.50%	17.83%	25.90%	5.29%
November	1,042	58.35%	41.65%	0.67%	14.68%	34.36%	20.35%	24.57%	5.37%
December	853	55.80%	44.20%	0.82%	16.65%	34.11%	20.63%	22.98%	4.81%

Figure S2: ALL Patient Clinical Complexity Level (PCCL) from January 2019 to December 2022

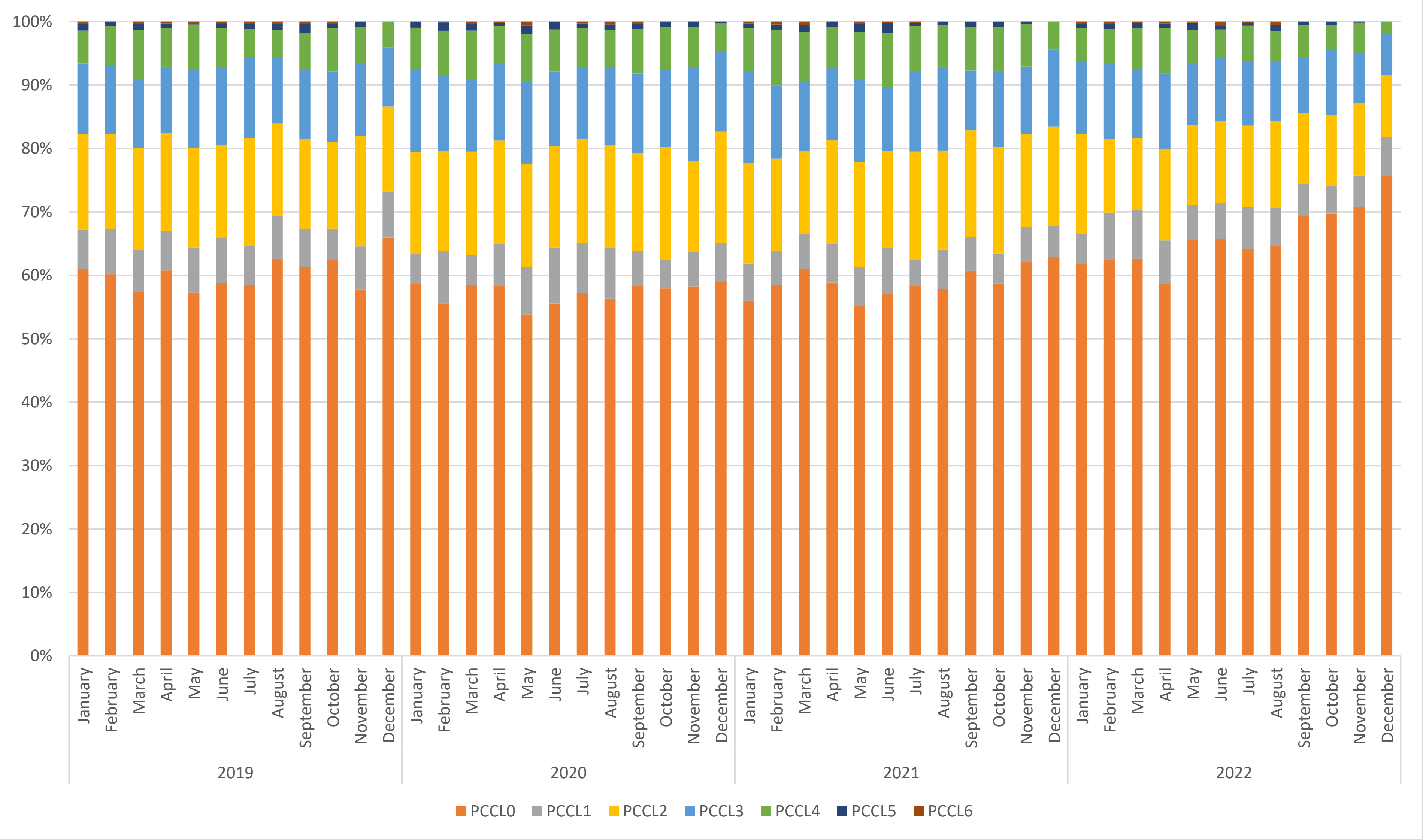

Figure S3: Number of ALL (C91.00) admissions by hospital size

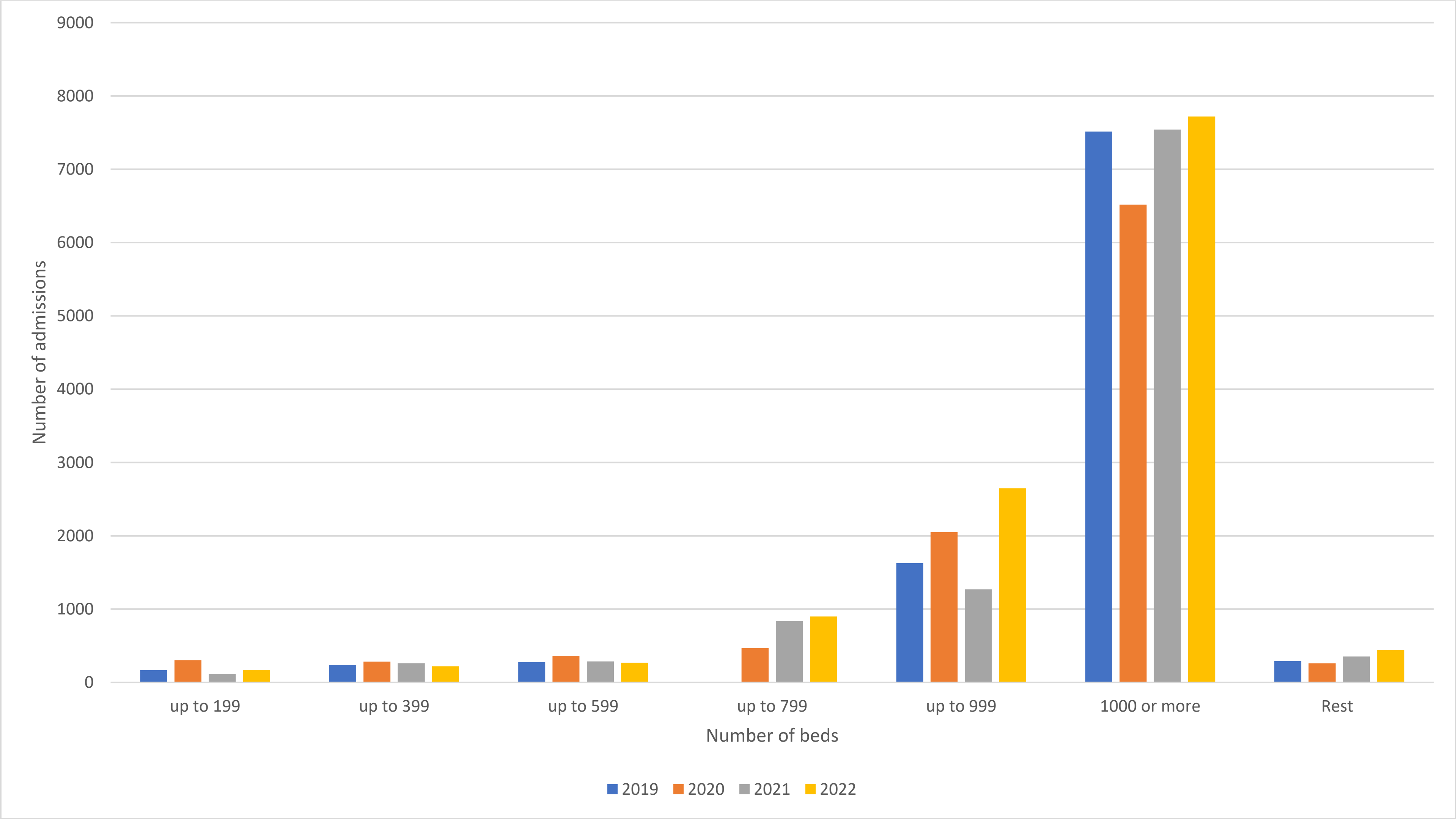

Figure S4: Share of ALL (C91.00) admissions by hospital size

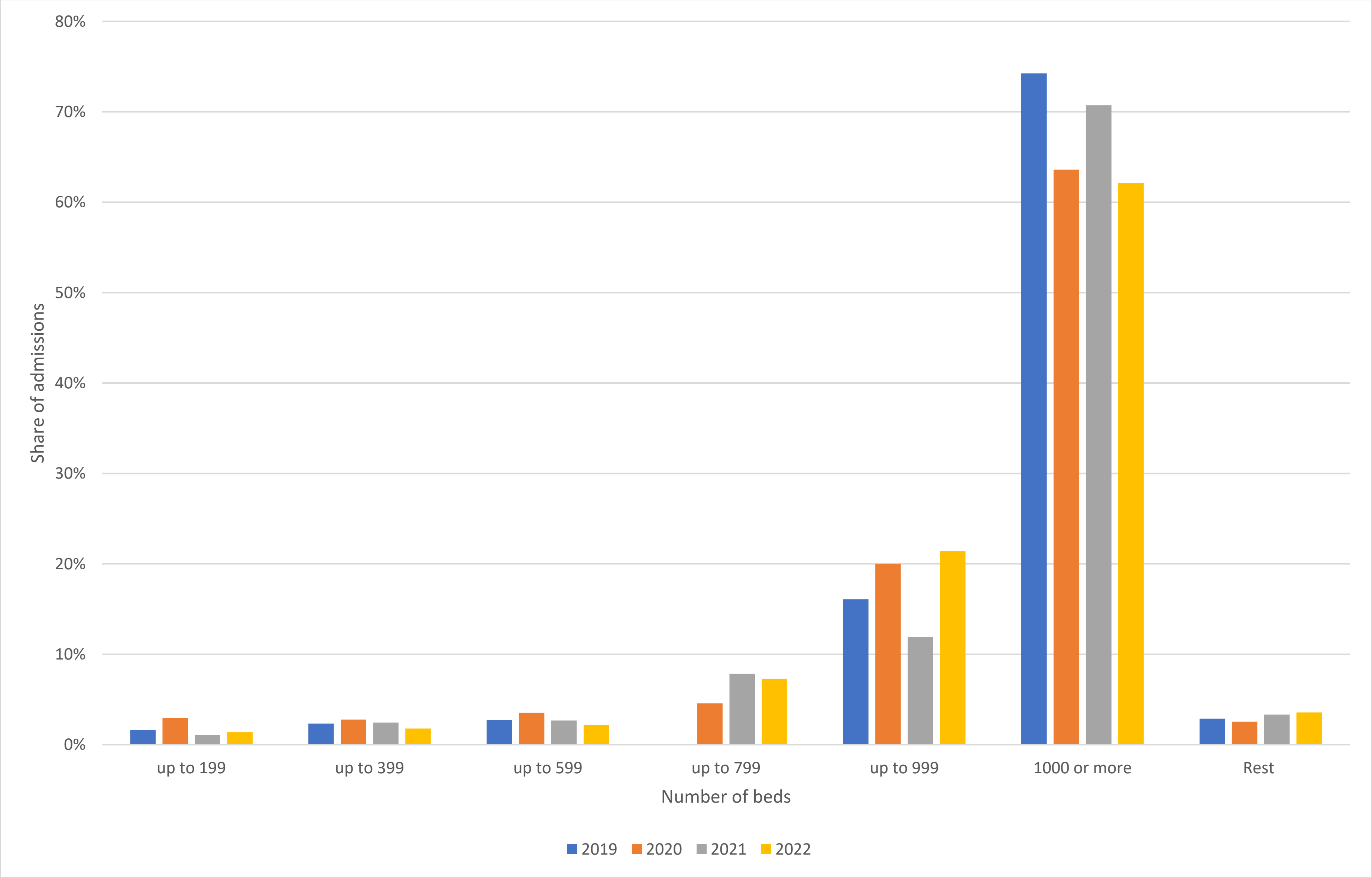

Section 4: Appendicitis/Appendectomy

Figure S5: Relative (%) change in monthly admissions compared to 2019 by level of complication, 2020-2022

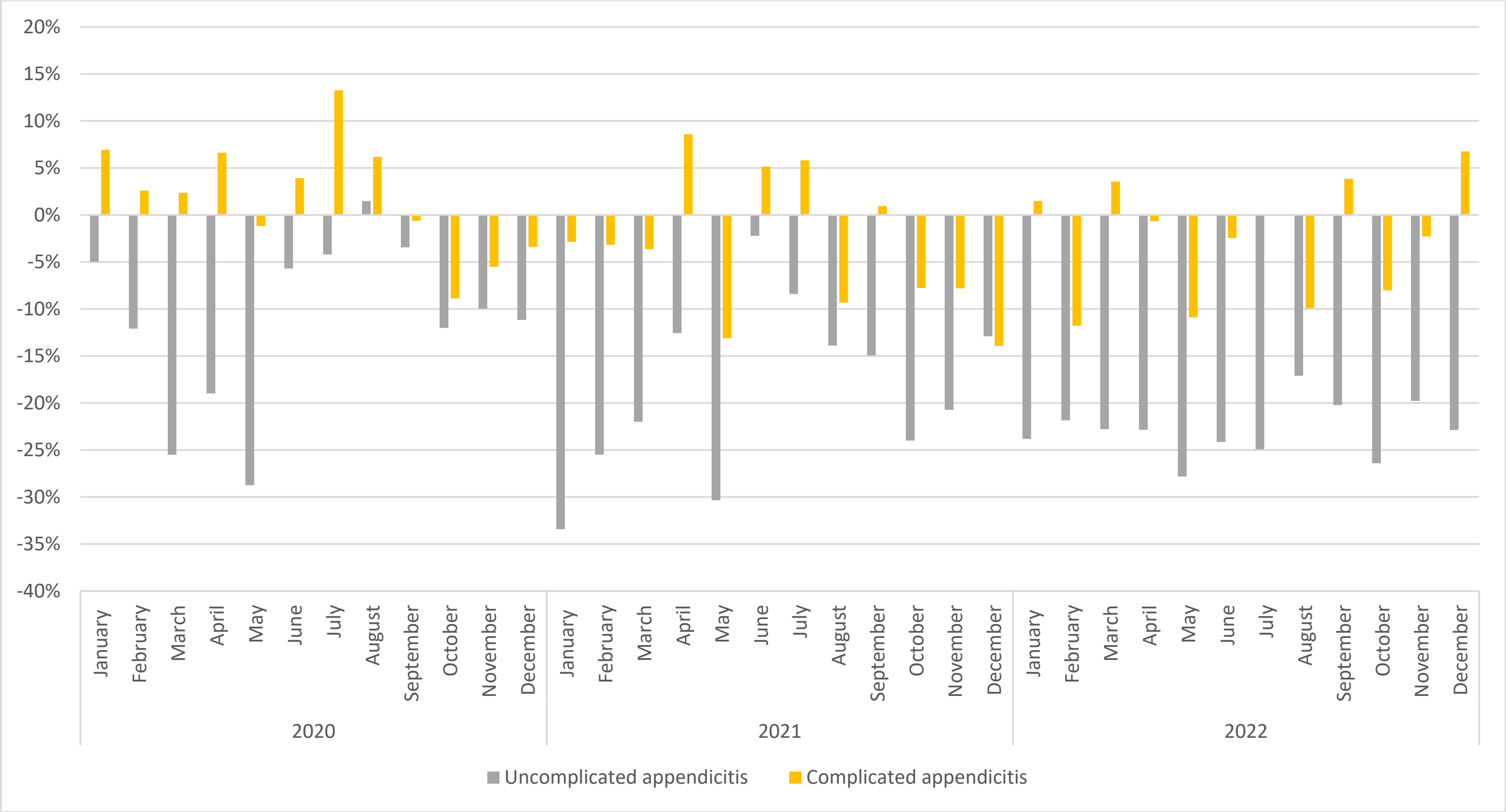

Figure S6: Number of Appendicitis admissions by hospital size

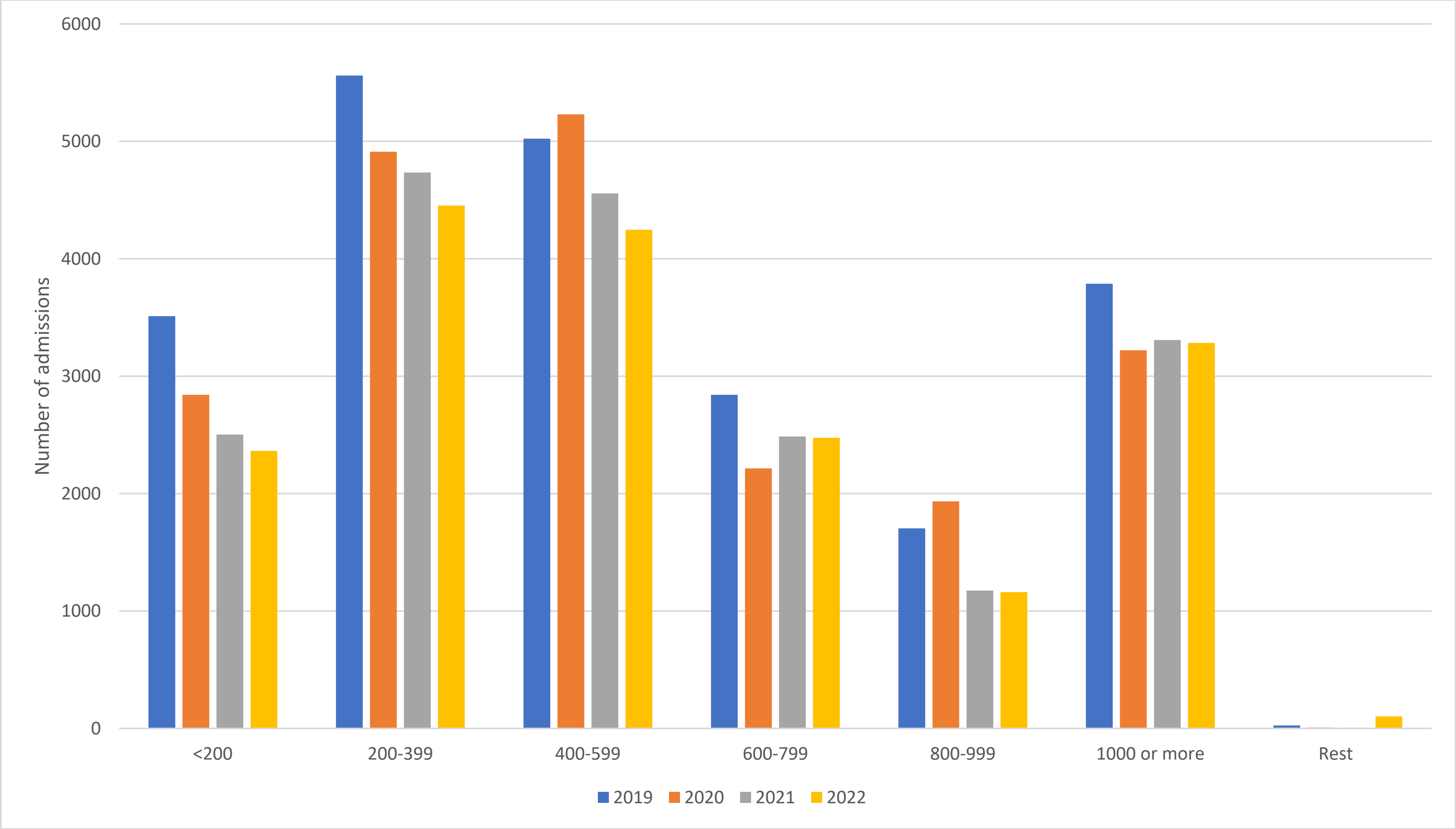

Figure S7: Share of appendicitis admissions by hospital size

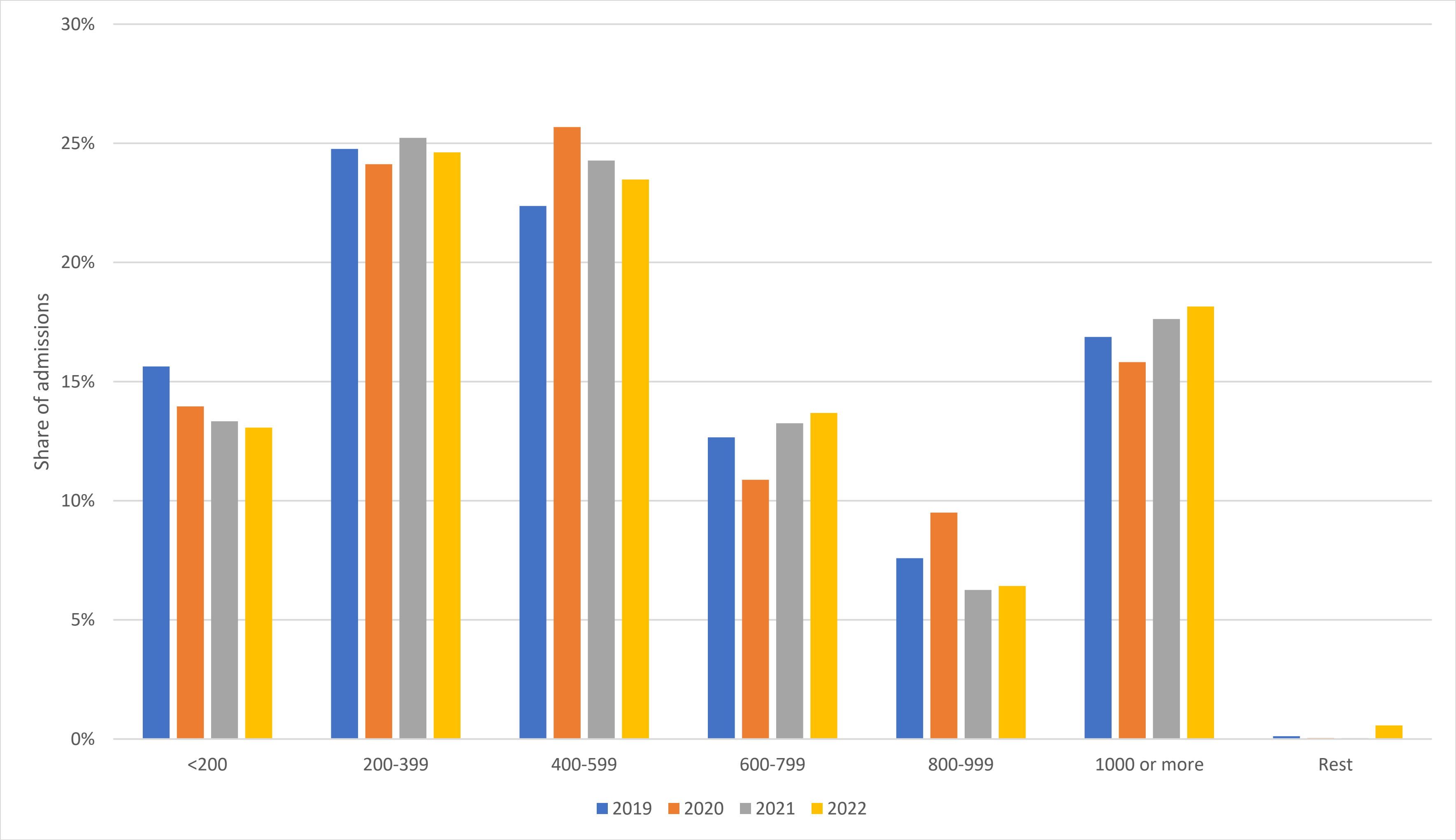

Section 5: Tonsillectomy/Adenoidectomy

Figure S8: Monthly relative (%) change in admissions who underwent tonsillectomy/adenoidectomy compared to 2019, 2020-2022

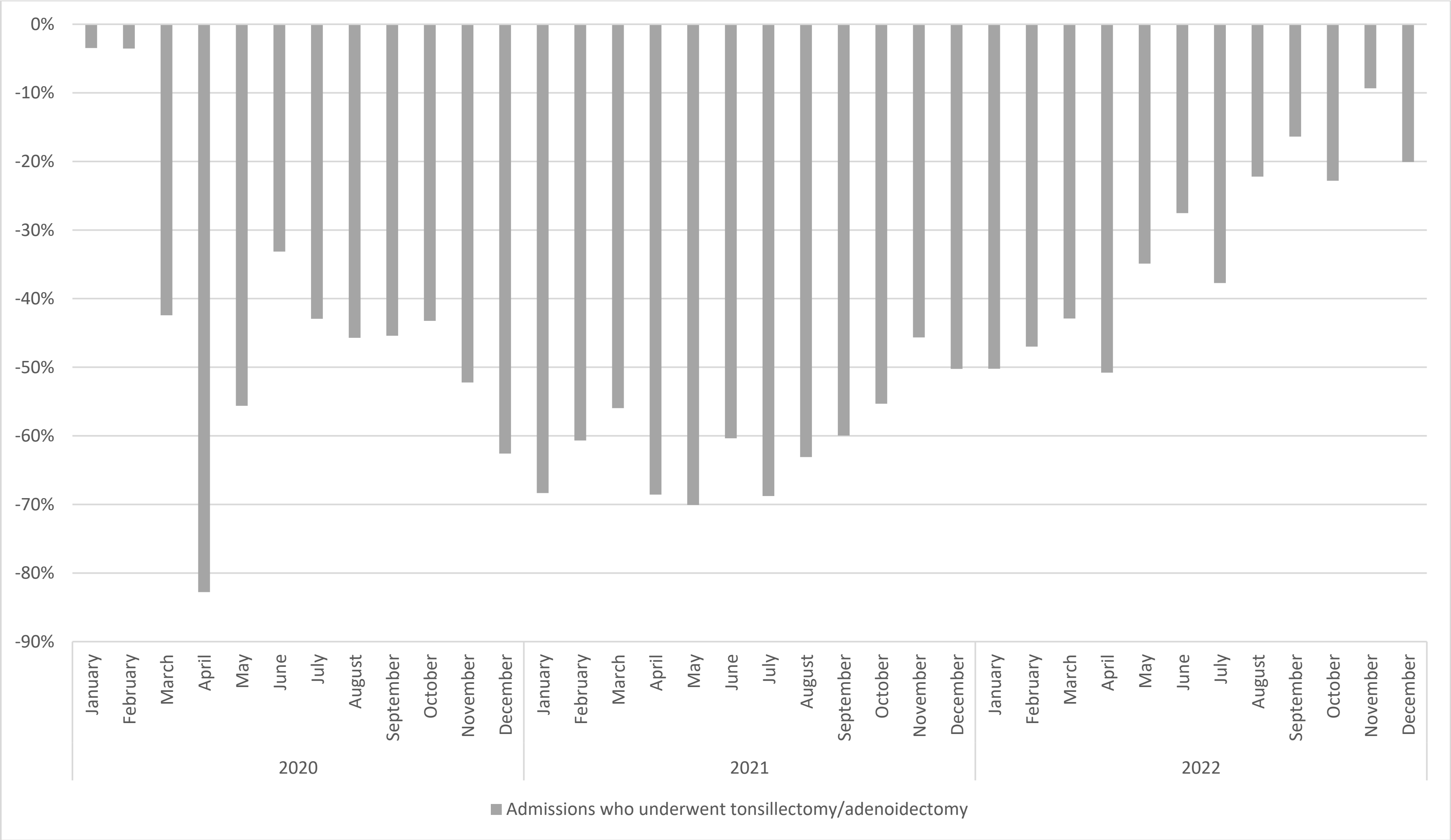

Figure S9: Number of tonsillectomy/adenoidectomy procedures by hospital size

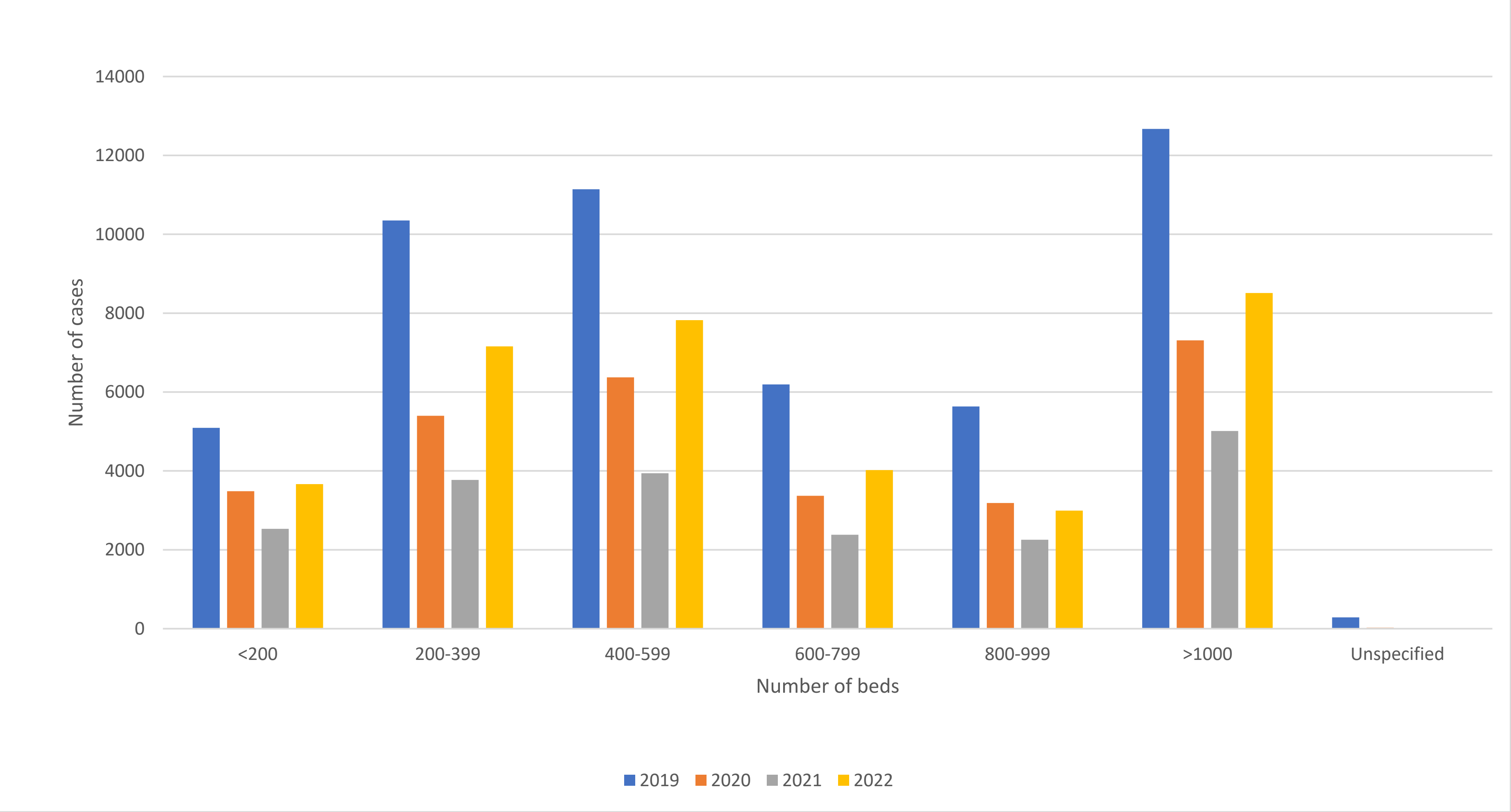

Figure S10: Share of tonsillectomy/adenoidectomy procedures by hospital size

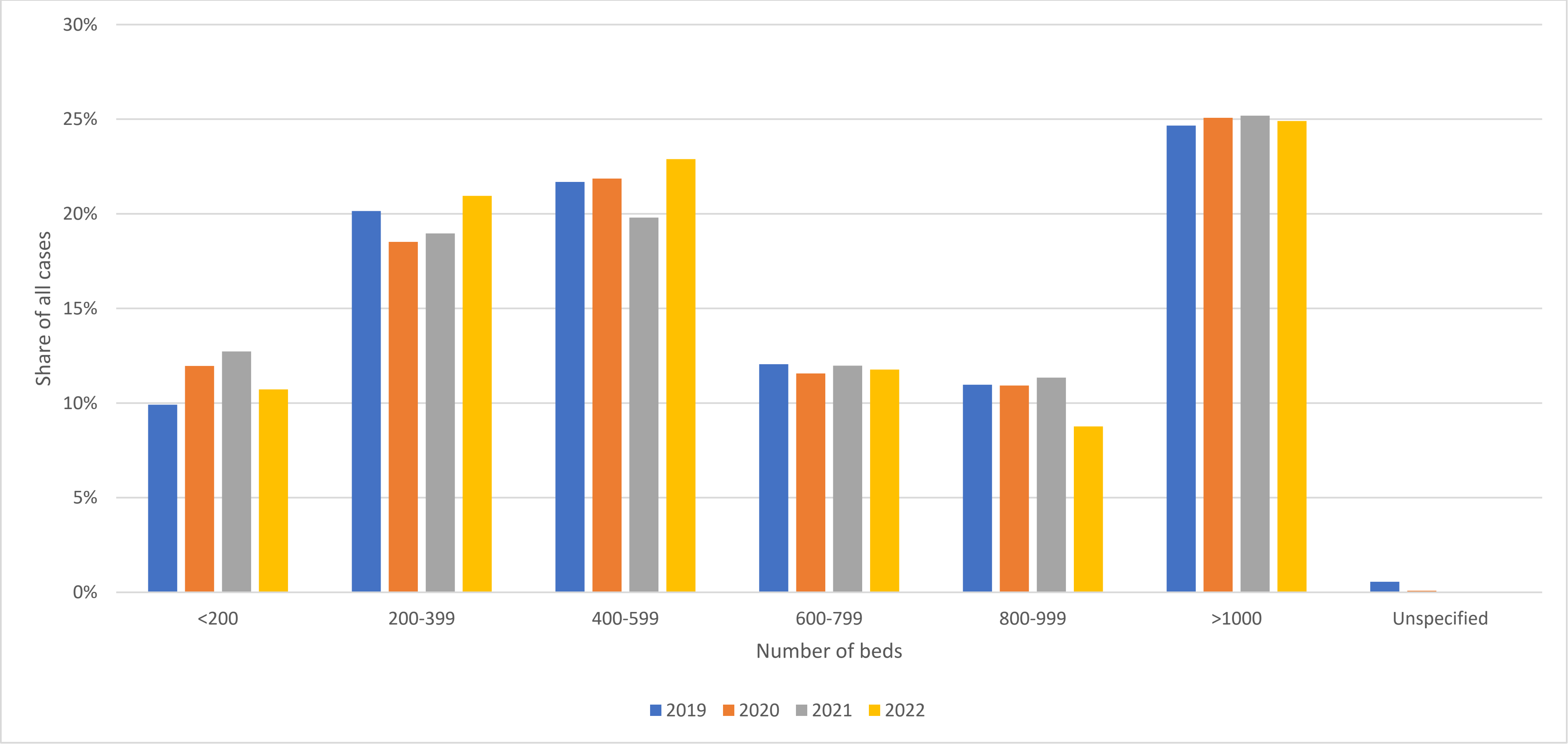

Supplement: Supplementary file 1 — Supplementary Material 1 [file 12913_2023_9929_MOESM1_ESM.pdf]
